# Comments on: prevalence and risk factors for tuberculosis and HIV coinfections in Kenyan prison inmates

**DOI:** 10.1186/s41182-024-00650-z

**Published:** 2024-10-31

**Authors:** Muhammad Hamza, ZIL-E-Huma Jalil, Abid Jan Abdul Sattar, Hamnah Sohail, Malik Olatunde Oduoye

**Affiliations:** 1 Department of Internal Medicine, Saidu Medical College, Swat, Pakistan; 2 Department of Internal Medicine, Sahiwal Medical College, Sahiwal, Pakistan; 3 Department of Research, The Medical Research Circle (MedReC), Goma, Democratic Republic of the Congo

**Keywords:** TB, HIV, Inmates, Malnutrition, Infection, Smoking

## Abstract

The study by Mwatenga et al. found a tuberculosis (TB) prevalence of 10.2% and an HIV prevalence of 19.1% among inmates, with all TB cases co-infected with HIV. Education level, smoking history, and substance use are key predictors of coinfections. Although informative, its single-centred design and reliance on sputum samples may be insufficient for some patient groups, potentially compromising diagnostic accuracy. Expanding the study to include several jails and employing more diagnostic procedures may increase reliability and the ability to generalize. The report also notes the lack of debate on broader socioeconomic variables and structural barriers to healthcare in jails, which are crucial to understanding inmates' health challenges. Overcrowding, malnutrition, and a poor healthcare system are examples of environmental factors that probably contribute to the spread of tuberculosis and make managing HIV more difficult. Additionally, not enough is known about the dietary habits of prisoners and other health conditions like diabetes or mental health, which may have an impact on the course of HIV and TB. Future studies should take these extra characteristics into account to create more comprehensive approaches to controlling HIV coinfections and TB in prison populations. This will help build more effective therapies.

Dear Editor,

This article aims to praise Mwatenga et al. on their detailed study, published in *Tropical Medicine and Health*, on the prevalence and associated variables of tuberculosis (TB) and HIV coinfections among inmates in a Kenyan jail [1]. This research gives crucial insights into a mostly under-studied group, emphasizing the necessity for focused treatments in correctional facilities.

The study’s findings, which found a TB prevalence of 10.2% and an HIV prevalence of 19.1%, with all TB patients co-infected with HIV, highlight a serious public health issue in prison settings [1]. The discovery of education level, smoking history, and illegal substance use as major predictors of these coinfections is particularly noteworthy [1]. These findings are consistent with prior research demonstrating that prisoners are at a higher risk of developing TB and HIV due to environmental and behavioural variables [2].

Although the study is exciting certain issues need to be addressed. The study's main limitation is that it is a single-centred study as mentioned by Mwatenga et al. [1]. One of the main drawbacks of single-centred research, like the one conducted in a Kenyan prison, is that it needs to be more generalizable. Because the study was limited to a single jail, its results might not apply to other locations, nations, or areas where variables such as local illness rates, healthcare standards, prison architecture, and inmate demographics might differ. Furthermore, the study's statistical power may be limited by the relatively small sample size, making it challenging to generalize findings to the larger jail population or other contexts. These limitations highlight the necessity of conducting a larger population study, such as expanding this research to include several prisons across Kenya or Sub-Saharan Africa, which could provide a more comprehensive picture of TB and HIV coinfections in correctional facilities. This would enable better generalization of the findings and aid in creating region-specific intervention methods.

Another limitation is that all participants were required to produce sputum (early morning and spot) samples on two consecutive days [1]. Expectorated sputum remains the standard sample for diagnosing pulmonary tuberculosis (PTB), however, some patients, like elderly adults, and extremely unwell HIV+ people, may be unable to expectorate sputum adequately, making PTB diagnosis challenging in these subpopulations [3]. The instructions given to these participants for sample collection (for example, coughing technique), although the quality of sputum samples can vary, might have not been followed exactly, potentially impacting the diagnostic accuracy of this study.

Samples were processed within 3 days and kept at 4 °C in the case of delay [1]. While storing samples at 4 °C is standard procedure for short-term storage, there may be variability in how well the cold chain is maintained. The literature reports that the viability of the M. tuberculosis complex was 80–94.4% when sputum was refrigerated at 4 °C [4, 5]. However, there are no details regarding the storage of these samples.

The wider social and environmental issues, such as poverty and subpar living circumstances outside of jail, which have an impact on the health of convicts, have rarely been discussed concerning the prevalence of TB and HIV coinfections in prison. Patients with HIV/TB coinfection frequently encounter behavioural, social, and economic obstacles to effective treatment in resource-constrained nations. These obstacles take the form of inadequate treatment literacy, lack of access to care, poverty, gender inequity, malnourishment, and stigmas associated with HIV in society [6]. As these are recognized risk factors for HIV and tuberculosis, these facts ought to be disclosed. Incorporating socioeconomic and environmental elements into the analysis of the risks and problems faced by inmates will lead to a more comprehensive understanding of the risks and challenges that prisoners face, highlighting the need for holistic public health strategies that extend beyond prison walls.

The structural obstacles that inmates have when trying to receive healthcare—such as TB and HIV testing, diagnosis, and treatment—are not sufficiently covered in the discussion. These obstacles can consist of a deficient infrastructure for healthcare, a shortage of medical professionals with the necessary training, and a shortage of medical supplies.

Malnutrition may exacerbate treatment outcomes for TB and MDR/RR-TB and contribute to the rise of TB medication resistance [7]. One established risk factor for HIV and tuberculosis is malnutrition. A person's immune system can be weakened by inadequate diet, increasing susceptibility to illnesses and decreasing recovery time. Regarding the convicts' dietary status, no information is available. We need to know how these inmates doing nutritionally.

Since TB is highly contagious and is made more contagious by environments that encourage poverty, overcrowding, and a lack of public health infrastructure, preventing the illness is still a global concern [8]. These circumstances are common in prisons by design, which can aid in the spread of TB. Information regarding the prison's state and ventilation should be made public to identify these risk factors.

Immune system-compromising conditions, such as diabetes mellitus (DM), which the World Health Organization (WHO) has identified as a major cause of TB [9], or chronic liver disease, can make people more susceptible to TB. Furthermore, as 42% of TB patients are thought to have mental health issues, mental health has a major impact on the disease as well [10]. For a better understanding of these risk factors for the convicts, information about these inmates that is not available must be provided.

Overall, this study significantly advances our knowledge of HIV and TB coinfections in prison populations. However, more multicenter studies keeping in mind the concerns expressed herein, are recommended to expand on these discoveries to give us a better insight into the prevalence and risk factors for TB/HIV confections in inmates.

## Data Availability

No datasets were generated or analysed during the current study.

## Declarations

### Provenance and peer review

Not commissioned, externally peer-reviewed.

### Declaration of generative Al and Al-assisted technologies in the writing process

There is nothing to disclose.
